# Fgf receptor 3 activation promotes selective growth and expansion of occipitotemporal cortex

**DOI:** 10.1186/1749-8104-4-4

**Published:** 2009-02-03

**Authors:** Rachel E Thomson, Peter C Kind, Nicholas A Graham, Michelle L Etherson, John Kennedy, Ana C Fernandes, Catia S Marques, Robert F Hevner, Tomoko Iwata

**Affiliations:** 1 Division of Cancer Sciences and Molecular Pathology, University of Glasgow, Beatson Laboratories for Cancer Research, Garscube Estate, Switchback Road, Glasgow, G61 1BD, UK; 2 Centre for Integrative Physiology, University of Edinburgh, Edinburgh, EH8 9XD, UK; 3 Department of Neurological Surgery and Pathology, University of Washington School of Medicine, Seattle, Washington, 98101-1304, USA

## Abstract

**Background:**

Fibroblast growth factors (Fgfs) are important regulators of cerebral cortex development. Fgf2, Fgf8 and Fgf17 promote growth and specification of rostromedial (frontoparietal) cortical areas. Recently, the function of Fgf15 in antagonizing Fgf8 in the rostral signaling center was also reported. However, regulation of caudal area formation by Fgf signaling remains unknown.

**Results:**

In mutant mice with constitutive activation of Fgf receptor 3 (Fgfr3) in the forebrain, surface area of the caudolateral cortex was markedly expanded at early postnatal stage, while rostromedial surface area remained normal. Cortical thickness was also increased in caudal regions. The expression domain and levels of Fgf8, as well as overall patterning, were unchanged. In contrast, the changes in caudolateral surface area were associated with accelerated cell cycle in early stages of neurogenesis without an alteration of cell cycle exit. Moreover, a marked overproduction of intermediate neuronal progenitors was observed in later stages, indicating prolongation of neurogenesis.

**Conclusion:**

Activation of Fgfr3 selectively promotes growth of caudolateral (occipitotemporal) cortex. These observations support the 'radial unit' and 'radial amplification' hypotheses and may explain premature sulcation of the occipitotemporal cortex in thanatophoric dysplasia, a human *FGFR3 *disorder. Together with previous work, this study suggests that formation of rostral and caudal areas are differentially regulated by Fgf signaling in the cerebral cortex.

## Background

The mammalian cerebral cortex consists of multiple cortical areas with specific functions, each of which connects to a specific area of the body and within the cortex [1]. Patterning and neurogenesis are two key events that influence cortical area formation. The cortical wiring is established based on the prospective area boundaries, the 'protomap', specified by combinations of transcription factors, such as Pax6 and Emx2, expressed in graded patterns in the cortical ventricular zone (VZ) [2-4]. Transcription factor expression gradients are modified by morphogens, expressed focally in the signaling centers during early forebrain development. Fgf8 is expressed in the rostral signaling center, and controls transcription factor expression gradients and area specification by its function in patterning. Concurrently, the cortex grows in size by controlled proliferation and differentiation of progenitor cells in the VZ and subventricular zone (SVZ). This process, neurogenesis, is also regulated by Fgf8, as well as by other Fgfs.

Fgf ligands comprise a family of 22 polypeptides that play a variety of roles during brain development [5]. Fgf8 and Fgf17 are expressed in the rostral-most region of the cortical primordium, and specify the frontal cortex and its subdivisions [6-9]. In addition, Fgf8 is shown to regulate the size of the forebrain, especially rostral regions [7,8]. Likewise, *Fgf2 *knockout mice showed significant reductions of neuronal density and cortical thickness in rostral areas, indicating its important role in neurogenesis [10-12]. Furthermore, a recent study of *Fgf15 *knockout mice revealed a function of Fgf15 in the rostral signaling center influencing patterning in a fashion opposite of Fgf8, suppressing proliferation, and promoting differentiation of cortical progenitors [13]. In contrast, no specific role for Fgfs has been found in regulating formation of caudal cortical areas.

The effects of Fgfs are mediated by the four high-affinity receptor tyrosine kinases, Fgfrs [14,15]. Signaling by Fgfrs was shown to be necessary for growth of rostral, as well as caudal, cortical regions [16]. However, current knowledge concerning the specific roles of individual receptors is limited, presumably due to functional redundancy. Nonetheless, some functions of Fgfrs have emerged. Forebrain-specific knockout of Fgfr1 (*Foxg1-Cre;Fgfr1*^*flox*/*flox*^) showed a loss of the olfactory bulb, mostly owing to a decrease in progenitor proliferation leading to a deficit in initial bulb evagination [17]. A subtle shift of Pax6 and Emx2 expressions also indicated an alteration in early patterning in this study. Changes in gene expression were more thoroughly assessed by microarray performed in developing cortex in this model at embryonic day (E)12.5, which confirmed the involvement of Fgfr1 signaling in the regulation of cortical patterning genes [18]. Dorso-ventral patterning defects were further demonstrated in a double knockout study, where deletion of both Fgfr1 and Fgfr3 resulted in a loss of neurogenesis in ventromedial regions, while deletion of both Fgfr1 and Fgfr2 led to a loss of ventral identities owing to dorsoventral patterning defects at E12.5 [19]. In addition, signaling via Fgfr1, but not Fgfr3, was shown to be required for growth of midline progenitor cell types at E14.5-E16.5 and formation of commissural axon tracts [20,21].

*In vitro *assays have shown that Fgfr3 responds highly to Fgf8/17, and to Fgf2 [15,22]. Fgfr3 is expressed in progenitor cells in the neuroepithelium from E9.5 and in the cortical VZ/SVZ throughout neurogenesis in mice [23], but is not expressed in postmitotic cortical plate neurons. Interestingly, Fgfr3 is expressed in a rostromedial-low, caudolateral-high gradient in the cortical primordium during E11.5-E13.5 [7,24-26]. *Fgfr3 *knockout mice show skeletal overgrowth and deafness owing to inner ear defects, indicating the role of Fgfr3 in skeletal growth and hearing [27]. However, the role of Fgfr3 in brain development has been uncertain. In particular, Fgfr3 was reported to have no definite role in cortical patterning [28]. Also, deletion of Fgfr3 did not aggravate the effects of either Fgfr1 or Fgfr2 deletion in the dorsal cortex and the midline [19,20]. However, the expected redundancy of Fgfr activities, and possible subtleties in the *Fgfr3*^-/-^brain phenotype, could mask Fgfr3 functions in cortical development.

A different perspective on the role of Fgfr3 in brain development has emerged from gain-of-function models. We previously showed that mice expressing a constitutively active mutant *Fgfr3 *allele displayed an enlarged cerebral cortex with increased cortical thickness and total cell number, mostly due to increased progenitor proliferation [26,29]. Furthermore, the increase in progenitor proliferation in the cortical VZ was graded along the rostrocaudal axis, with the highest effect in caudal region during the early stages of neurogenesis at E11-E13 [26]. Downstream activation of MAPK in E11.5 temporal cortex was largely responsible for this effect. These conditional knock-in mouse models carry mutations substituting the amino acid Lys644 to glutamic acid (K644E) and to methionine (K644M) [30,31]. The mutant allele is knocked-in to the endogenous *Fgfr3 *locus, allowing expression of the mutant allele in a fashion reflecting normal Fgfr3 expression domains and levels. K644 mutations highly activate Fgfr3 signaling in terms of kinase activity and autophosphorylation *in vitro *[31-33]. BaF3, mouse pro-B cell line expressing Fgfr3 with this mutation, shows a mitogenic response both in the absence of ligand (approximately 25% of full response) and in a concentration-dependent manner (an increasing response by a further addition of ligand) with an overall 2- to 10-fold increase of response compared to control [32].

The corresponding kinase-domain mutations in human *FGFR3 *are known to cause a severe and fatal form of achondroplasia, known as thanatophoric dysplasia (TD; OMIM#187601). The cerebral neocortex in TD is markedly enlarged (megalencephaly) and excessively convoluted, especially in temporal and occipital lobes, where sulcation begins prematurely [34]. In addition, TD brains show severe hippocampal dysplasia with rudimentary dentate gyrus, as well as numerous abnormalities of the brainstem and cerebellum [34]. Therefore, the study of Fgfr3 function presents a unique opportunity whereby its biological significance in cerebral cortical development can be directly compared in mice and humans.

In the present study, we hypothesized that Fgfr3 may play a role in formation of cortical areas by regulating patterning in addition to progenitor proliferation and focused to analyze cortical area formation and changes in cell cycle parameters. We found that mice carrying biochemically activating mutations in Fgfr3 presented a massive enlargement of the caudolateral cortex surface area in early postnatal stage, with relatively little change in the rostral cortex. The expression of transcription factors remained mostly normal, indicating that patterning of the cortex is influenced little by Fgfr3 activation. In contrast, cumulative bromodeoxyuridine (BrdU) labeling revealed that the length of the G1 phase (T_G1_) was 1.7 hours shorter in progenitors of the caudal mutant VZ compared to the wild type at E12.5. Therefore, the expansion of caudal cortex surface area likely reflected excessive proliferation of radial glia at early stages of neurogenesis. Finally, cortical thickness was increased in caudal areas of the *Fgfr3 *mutant mice, which may be explained by a significant increase of intermediate neuronal progenitors at late stages (E18.5), indicating a prolongation of neurogenesis. In summary, this study reveals a unique perturbation of cortical area formation with selective expansion of the caudal cortex surface area, caused by alteration of Fgf signaling along the Fgfr3 gradient.

## Results

### Selective expansion of caudolateral cortical areas in the Fgfr3 mutant mice

We first addressed whether the change in Fgf signaling along the graded expression of Fgfr3 could influence formation of cortical areas, using mouse models with the gain-of-function, kinase domain mutations targeted in the *Fgfr3 *gene locus [30,31]. These mice were originally generated to represent the severe skeletal dysplasia. Early lethality associated with the disease needed to be prevented in order to successfully propagate the animal model. It is known that the presence of an exogenous fragment in the *Fgfr3 *locus suppresses transcription of the *Fgfr3 *allele [35]. Taking advantage of this, we designed the homologous recombination so that the *neo *gene flanked by the loxP sequences was inserted in intron 10 located near exon 15 containing the mutation site (*Fgfr3*^+/*K644Eneo*^). Upon crosses with mice that express Cre recombinase, the *neo *gene is removed and the allele with *Fgfr3 *mutation is transcribed (for example, *EIIa-Cre;Fgfr3*^+/*K644E*^) [30,31].

Formation of the cortical areas can be detected by analyzing the projection of thalamocortical axons to the cortex at postnatal week 1 in mice [4]. Ubiquitous expression of the K644E mutation heterozygously (*EIIa-Cre;Fgfr3*^+/*K644E*^) leads to lethality within postnatal day 1 (P1), possibly owing to the skeletal phenotype [30], and is unsuitable for postnatal analysis of cortical areas. We therefore tested the survival of mice with K644E and K644M mutations [31] by crossing with *Nestin- *and *Foxg1-Cre *mice, which allows expression of Cre recombinase preferentially in the brain. *Nestin-Cre *[36] drives recombination in the central nervous system starting from E11. In contrast, *Foxg1-Cre *[37] allows recombination starting from E8.5 in more limited regions, mainly in the forebrain, although some other regions such as facial and head ectoderm, mid-hindbrain junction, and pharyngeal pouches are also shown to express Cre recombinase. As the axonal growth from the diencephalon would affect development of the neocortex, use of *Foxg1-Cre *is advantageous. The result of the crosses showed that, despite the lack of skeletal abnormalities, the *Foxg1-Cre;Fgfr3*^+/*K644E *^offspring died within P1 (n > 8) from unknown causes. In contrast, *Nestin-Cre;Fgfr3*^+/*K644E *^and *Foxg1-Cre;Fgfr3*^+/*K644M *^offspring survived for more than 3 weeks (n > 10 each genotype). We therefore analyzed area formation using these lines.

**Figure 1:**
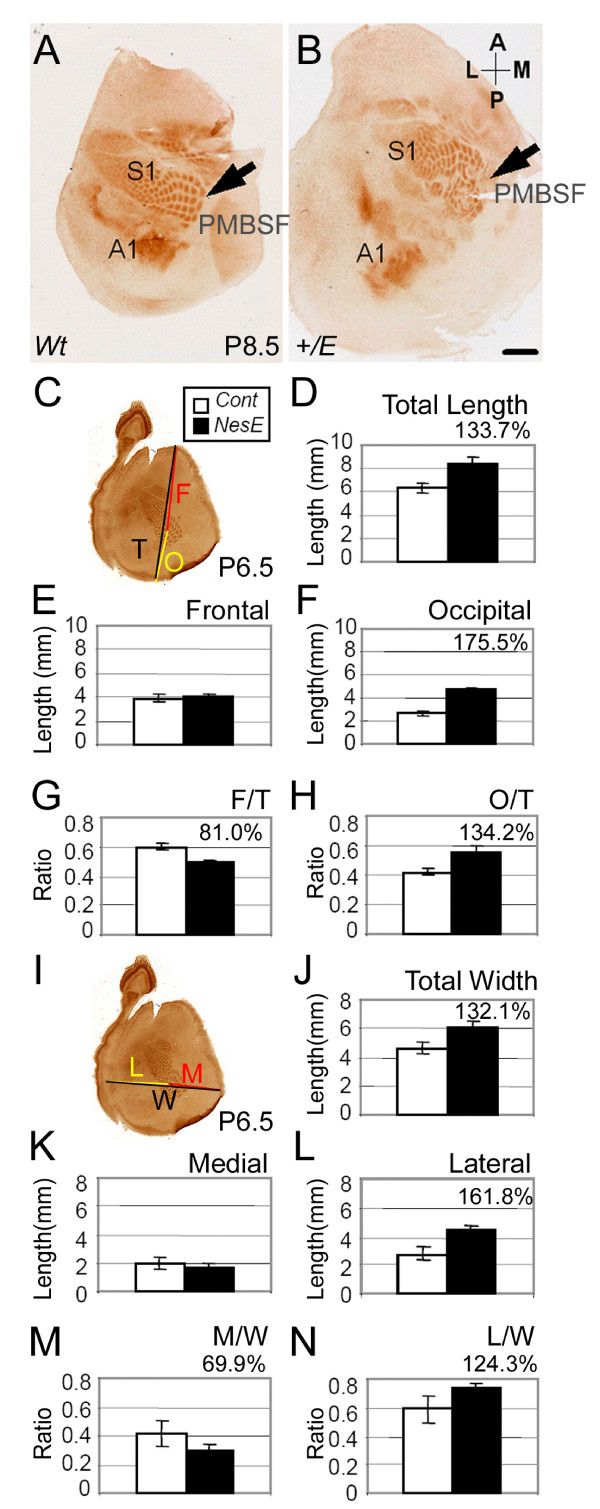
**Rostral shift of cortical areas in *Nestin-Cre;Fgfr3*^+/*K644E*^**. **(a, b) **Cortical areas were visualized in tangential sections of the flattened cortex of *Nestin-Cre;Fgfr3*^+/*K644E *^(b) and wild-type (Wt) littermates (a) by immunohistochemistry for the serotonin transport protein 5-hydroxy-tryptamine (5-HTT). In (a, b), cortical hemispheres at postnatal day (P)8.5 were dissected so that the primary somatosensory area (S1), including the posteromedial barrel subfield (PMBSF), was clearly displayed. Irregularity in the barrel pattern was observed in *Nestin-Cre;Fgfr3*^+/*K644E *^(b, arrow). **(c) **Position of the PMBSF was quantified by cytochrome C oxidase histochemistry performed at P6.5, using C4 subfield as a reference point, and presented as a frontal (F) and occipital (O) ratio to the total cortical length (T). **(d-f) **Measurements showed an increase in total and occipital length, while the frontal length remained similar. **(g, h) **The F/T and O/T length ratios clearly show that the PMBSF was rostrally shifted in *Nestin-Cre;Fgfr3*^+/*K644E*^. **(i) **Position of the PMBSF was quantified for the total (W), medial (M) and lateral (L) lengths. **(j-l) **The total and lateral lengths were increased in *Nestin-Cre;Fgfr3*^+/*K644E *^compared to wild type, while the medial length was similar. **(m, n) **The M/W and L/W ratios show that the PMBSF was medially shifted in *Nestin-Cre;Fgfr3*^+/*K644E*^. Scale bar: 1 mm in (a, b).

**Figure 2:**
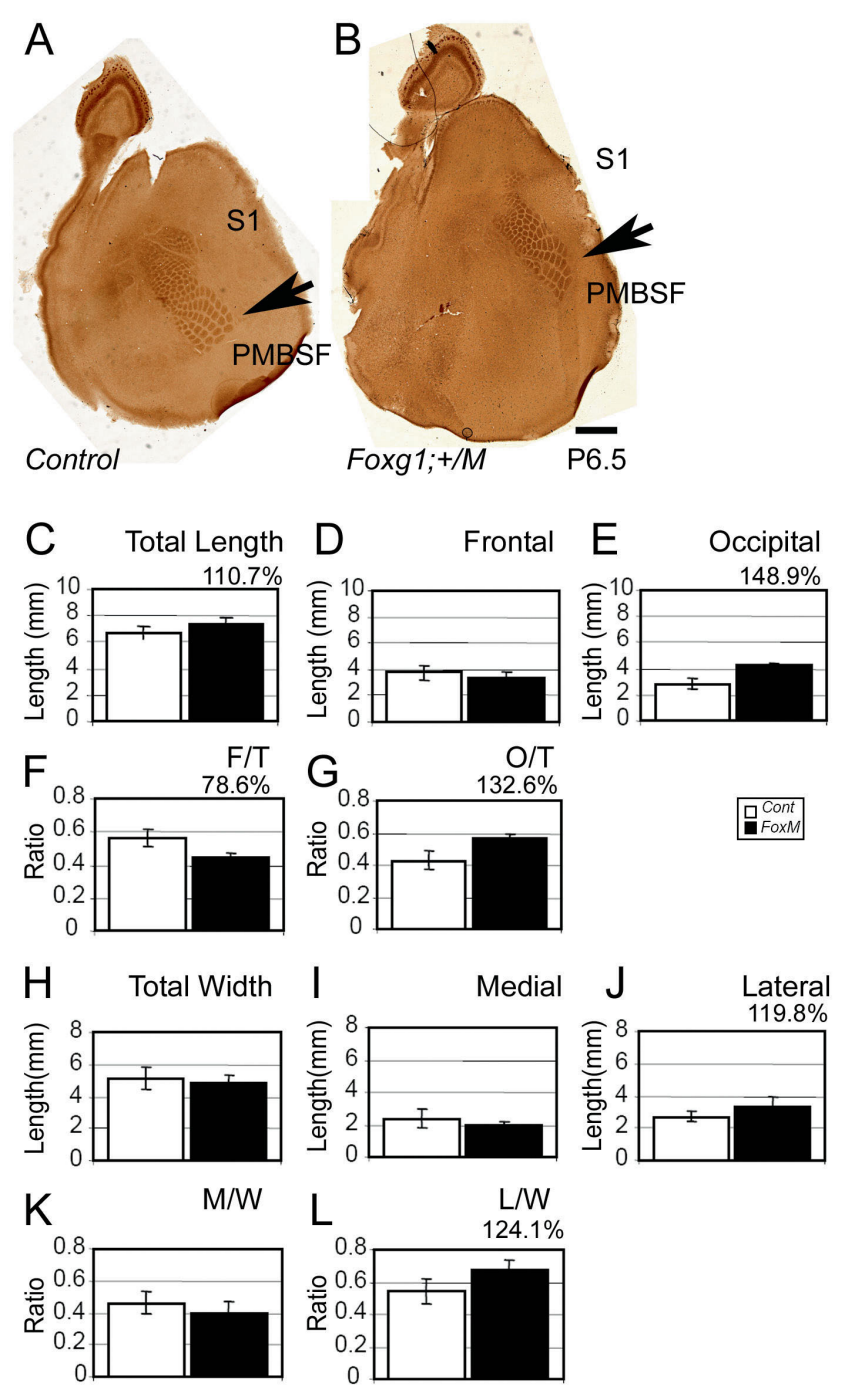
**Rostral shift of cortical areas in *Foxg1-Cre;Fgfr3*^+/*K644M*^**. **(a, b) **Formation of the cortical areas was analyzed in the flattened cortex of *Foxg1-Cre;Fgfr3*^+/*K644M *^(b) and control (a) at P6.5 by cytochrome C oxidase histochemistry. **(c-e) **Measurements show an increase in total and occipital lengths of the cortex, while the frontal length remained similar. **(f, g) **The F/T and O/T length ratios clearly show that the PMBSF was rostrally shifted in *Foxg1-Cre;Fgfr3*^+/*K644M*^. **(h-j) **The lateral length of the cortex was increased in *Foxg1-Cre;Fgfr3*^+/*K644M *^compared to control, while no significant difference was observed in the total and medial lengths. **(k, l) **The L/W ratio shows that the PMBSF was medially shifted in *Foxg1-Cre;Fgfr3*^+/*K644M*^. Scale bar: 1 mm in (a, b).

Cortical area formation was analyzed in early postnatal mice by immunohistochemistry for the serotonin transport protein 5-hydroxy-tryptamine (5-HTT), which detects thalamocortical axon terminals, as well as cytochrome C oxidase histochemistry, which visualizes the high cellular metabolism associated with synaptic activities [6,38,39]. Areas including the primary somatosensory (S1) and auditory (A1) areas were successfully visualized in *Nestin-Cre;Fgfr3*^+/*K644E *^(Figure 1a, b) and *Foxg1-Cre:Fgfr3*^+/*K644M *^(Figure 2a, b) in early postnatal stages. Surprisingly, irregularity of the S1 shape was observed in *Nestin-Cre;Fgfr3*^+/*K644E *^(Figure 1b); this was observed in all *Nestin-Cre;Fgfr3*^+/*K644E *^samples analyzed (n > 14), although the pattern of irregularity was not obviously consistent in each sample. This phenotype was not present in *Foxg1-Cre;Fgfr3*^+/*K644M *^cortices (n > 9; Figure 2b). The whisker pads of both *Nestin-Cre;Fgfr3*^+/*K644E *^and *Foxg1-Cre;Fgfr3*^+/*K644M *^animals were normal (data not shown). The cause of this particular phenotype in *Nestin-Cre;Fgfr3*^+/*K644E *^is likely to be attributed to changes in the thalamus and brainstem, where *Foxg1-Cre *does not drive expression of the mutant Fgfr3.

The position of S1 appeared to be rostrally shifted (relative to the rostral and caudal poles) in *Nestin-Cre;Fgfr3*^+/*K644E *^in comparison to wild-type littermates due to surface area expansion, which was particularly obvious in caudal regions (Figure 1a, b). A similar relative rostral shift of S1 with caudal enlargement was observed in *Foxg1-Cre;Fgfr3*^+/*K644M *^(Figure 2a, b). This phenotype was evident in all samples analyzed (n > 14 and 9 in *Nestin-Cre;Fgfr3*^+/*K644E *^and *Foxg1-Cre:Fgfr3*^+/*K644M*^, respectively). The appearance of the 'rostral shift' could result from selective reduction of rostral surface area or selective expansion of caudal area, or both. To address these possibilities, we quantitatively analyzed area location of the posteromedial barrel subfield (PMBSF) following previously established methods [39,40] at P6.5 (Figure 1c, i and Table 1). The total and occipital lengths of the cortex were increased by 33.7% and 75.5%, respectively, in *Nestin-Cre;Fgfr3*^+/*K644E *^compared to wild type, while the frontal length remained similar (Figure 1d–f and Table 1). As a result, the ratio of frontal-to-total length (F/T) was decreased by 19.0%, while that of occipital-to-total length (O/T) was increased by 34.2% (Figure 1g–h and Table 1), confirming the appearance of an overall 'rostral shift' in the *Nestin-Cre;Fgfr3*^+/*K644E *^cortex. We also compared mediolateral positions of the PMBSF. The total and lateral widths of the *Nestin-Cre;Fgfr3*^+/*K644E *^cortex were 32.1% and 61.8% longer than wild type, respectively, while the medial width did not change significantly (Figure 1j–l and Table 1). The ratio of the medial-to-total width (M/W) was decreased by 30.1%, while the lateral-to-total width (L/W) was increased by 24.3%, indicating a relative 'medial shift' (Figure 1m–n and Table 1). A similar change in area dimensions was observed and confirmed by quantitative analysis in the *Foxg1-Cre;Fgfr3*^+/*K644M *^cortices (Figure 2 and Table 2).

**Table 1:** Summary of measurements of the PMBSF position in *Nestin-Cre;Fgfr3*^+/*K644E*^

Measurement	Cont ± SD	n	*Nes+*/*E *± SD	n	%Cont	*P*-value
**Rostrocaudal**						
Total length (T) (mm)	6.38 ± 0.42	10	8.52 ± 0.48	5	133.7	5.3 × 10^-5^
Frontal length (F) (mm)	3.85 ± 0.30	10	4.08 ± 0.23	4	106.1	NS
Occipital length (O) (mm)	2.66 ± 0.24	10	4.67 ± 0.24	4	175.5	1.5 × 10^-5^
Ratio F/T	0.60 ± 0.024	10	0.49 ± 0.019	4	81.0	3.9 × 10^-5^
Ratio O/T	0.42 ± 0.023	10	0.56 ± 0.036	4	134.2	1.7 × 10^-3^
**Lateromedial**						
Total width (W) (mm)	4.60 ± 0.38	10	6.08 ± 0.38	5	132.1	8.9 × 10^-5^
Medial length (M) (mm)	1.94 ± 0.46	10	1.75 ± 0.15	4	90.2	NS
Lateral length (L) (mm)	2.73 ± 0.50	10	4.42 ± 0.40	4	161.8	2.9 × 10^-4^
Ratio M/W	0.42 ± 0.089	10	0.29 ± 0.041	4	69.9	3.6 × 10^-3^
Ratio L/W	0.59 ± 0.096	10	0.74 ± 0.038	4	124.3	1.6 × 10^-3^

Measurements were taken, as indicated in Figure 1c,i, to determine the rostrocaudal and lateromedial positions of the posteromedial barrel subfield (PMBSF) in *Nestin-Cre;Fgfr3*^+/*K644E *^(*Nes+*/*E*) and its littermate control (Cont) cortices at postnatal day 6.5. Results are presented in the graph in Figure 1d-g (rostrocaudal) and 1j-n (lateromedial positions). SD, standard deviation.

**Table 2:** Summary of measurements of the PMBSF position in *Foxg1-Cre;Fgfr3*^+/*K644M*^

Measurement	Cont ± SD	n	*Fox+*/*M *± SD	n	%Cont	*P*-value
**Rostrocaudal**						
Total length (T) (mm)	6.70 ± 0.51	7	7.42 ± 0.53	9	110.7	0.017
Frontal length (F) (mm)	3.75 ± 0.50	7	3.30 ± 0.44	9	88.0	NS
Occipital length (O) (mm)	2.84 ± 0.38	7	4.23 ± 0.24	9	148.9	1.0 × 10^-5^
Ratio F/T	0.56 ± 0.055	7	0.44 ± 0.032	9	78.6	8.0 × 10^-4^
Ratio O/T	0.42 ± 0.061	7	0.57 ± 0.023	9	132.6	4.6 × 10^-4^
**Lateromedial**						
Total width (W) (mm)	5.09 ± 0.70	7	4.86 ± 0.52	9	95.5	NS
Medial length (M) (mm)	2.37 ± 0.58	7	1.90 ± 0.26	9	80.2	NS
Lateral length (L) (mm)	2.73 ± 0.28	7	3.27 ± 0.61	9	119.8	0.038
Ratio M/W	0.46 ± 0.072	7	0.39 ± 0.074	9	84.8	NS
Ratio L/W	0.54 ± 0.073	7	0.67 ± 0.068	9	124.1	4.3 × 10^-3^

Measurements were taken, as indicated in Figure 1c,i, to determine the rostrocaudal and lateromedial positions of the posteromedial barrel subfield (PMBSF) in *Foxg1-Cre;Fgfr3*^+/*K644M *^(*Fox+*/*M*) and its littermate control (Cont) cortices at postnatal day 6.5. Results werare presented in the graph in Figure 2c-g (rostrocaudal) and 2h-l (lateromedial positions). SD, standard deviation.

Taken together, in the presence of the Fgfr3 kinase domain mutation, the surface area of the caudal and lateral cortex was particularly enlarged, while that of the rostral and medial areas showed little change, leading to an overall appearance of relative 'shifts' in rostral and medial directions towards the domain of less Fgfr3 expression.

### Cortical patterning was unchanged in the Fgfr3 mutant cortex

Positioning of cortical areas along the rostrocaudal and lateromedial axes is initially determined by patterning processes occurring early in cortical development. We next studied whether activation of Fgfr3 perturbs the expression of Fgf8 in the rostral signaling center at the initial patterning stage (E9.5) and those of cortical transcription factor gradients formed at E11.5-E13.5. For the analysis of embryonic brains, we used a model in which the Fgfr3 mutation is ubiquitously expressed (*Ella-Cre;Fgfr3*^+/*K644E*^) [26,29]. Use of *EIIa-Cre *is advantageous as, under the cross with this line, a ubiquitous promoter, *EIIa*, drives Cre recombination from the one-cell zygote stage. In the conditional knock-in line, expression of the mutant Fgfr3 allele remains under the transcriptional regulation of the endogenous Fgfr3 promoter, and as such its expression faithfully reflects the spaciotemporal expression of the endogenous Fgfr3 in *Ella-Cre;Fgfr3*^+/*K644E*^.

**Figure 3:**
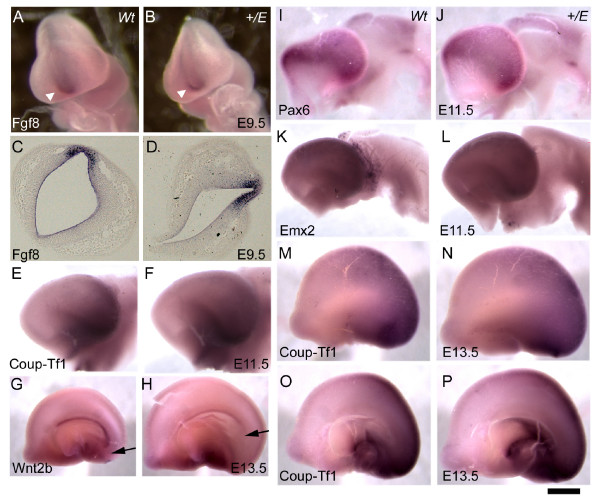
**Little change in gene expression patterning in the *EIIa-Cre;Fgfr3*^+/*K644E *^cortical primordium**. **(a-d) **Expression of Fgf8 was examined by whole mount *in situ *hybridization at E9.5. Focal expression of Fgf8 in the anterior neural ridge was observed as expected (white arrowhead) in wild type (Wt) (a, c), and a similar expression domain and level of Fgf8 was observed in *EIIa-Cre;Fgfr3*^+/*K644E *^(b, d). **(e, f, m-p) **Coup-Tf1 was expressed similarly in wild type (e, m, o) and *EIIa-Cre;Fgfr3*^+/*K644E *^(f, n, p) at E11.5 (g, h) and E13.5 (m-p). Panels (o, p) are medial views of (m, n), respectively. **(g, h) **Wnt2b marked the caudal limit of the cortical hem (arrows), indicating a selective expansion of the caudal region in *EIIa-Cre;Fgfr3*^+/*K644E*^. Note the overall reduction in expression level of Wnt2b in (h). No obvious difference was observed in expression of Pax6 at E11.5 **(i, j) **and Emx2 at E11.5 **(k, l) **between the wild-type (i, k) and *EIIa-Cre;Fgfr3*^+/*K644E *^samples (j, l). Results presented here are representatives of at least three independent hybridizations (n > 3). Scale bars: 250 μm in (a, b); 100 μm in (c, d); 400 μm in (e, f, i-l); 800 μm in (g, h, m-p).

Whole mount *in situ *hybridization showed no obvious differences in the expression domain or levels of Fgf8 in wild type and *Ella-Cre;Fgfr3*^+/*K644E *^at E9.5 (n > 6; Figure 3a–d). No obvious change was detected in the expression of Pax6 and Emx2 (Figure 3i–l), or Coup-Tf1 at E11.5 and E13.5 (n > 3; Figure 3e, f, m–p). Expression of Coup-Tf1 was also examined at E12.5 and E14.5 on sagittal sections [26], where a somewhat less steep gradient, possibly owing to an increase in expression at the rostral region, was observed. The presence of subtle changes in expressions of these factors cannot be excluded, but more precise quantitative methods are required for their detection.

An interesting change was observed in the expression of a cortical hem marker, Wnt2b [41]. Unlike in wild-type embryos, Wnt2b expression did not extend to the most caudal region of the cortical primordium in *EIIa-Cre;Fgfr3*^+/*K644E *^(Figure 3g, h). This could indicate an enlargement of the caudal region in *EIIa-Cre;Fgfr3*^+/*K644E*^. Furthermore, the overall level of Wnt2b expression appeared to be reduced, implicating Fgfr3 activity in a perturbation of cortical hem properties.

### Shorter cell cycle length in the caudal Fgfr3 mutant cortex at E12.5

**Figure 4:**
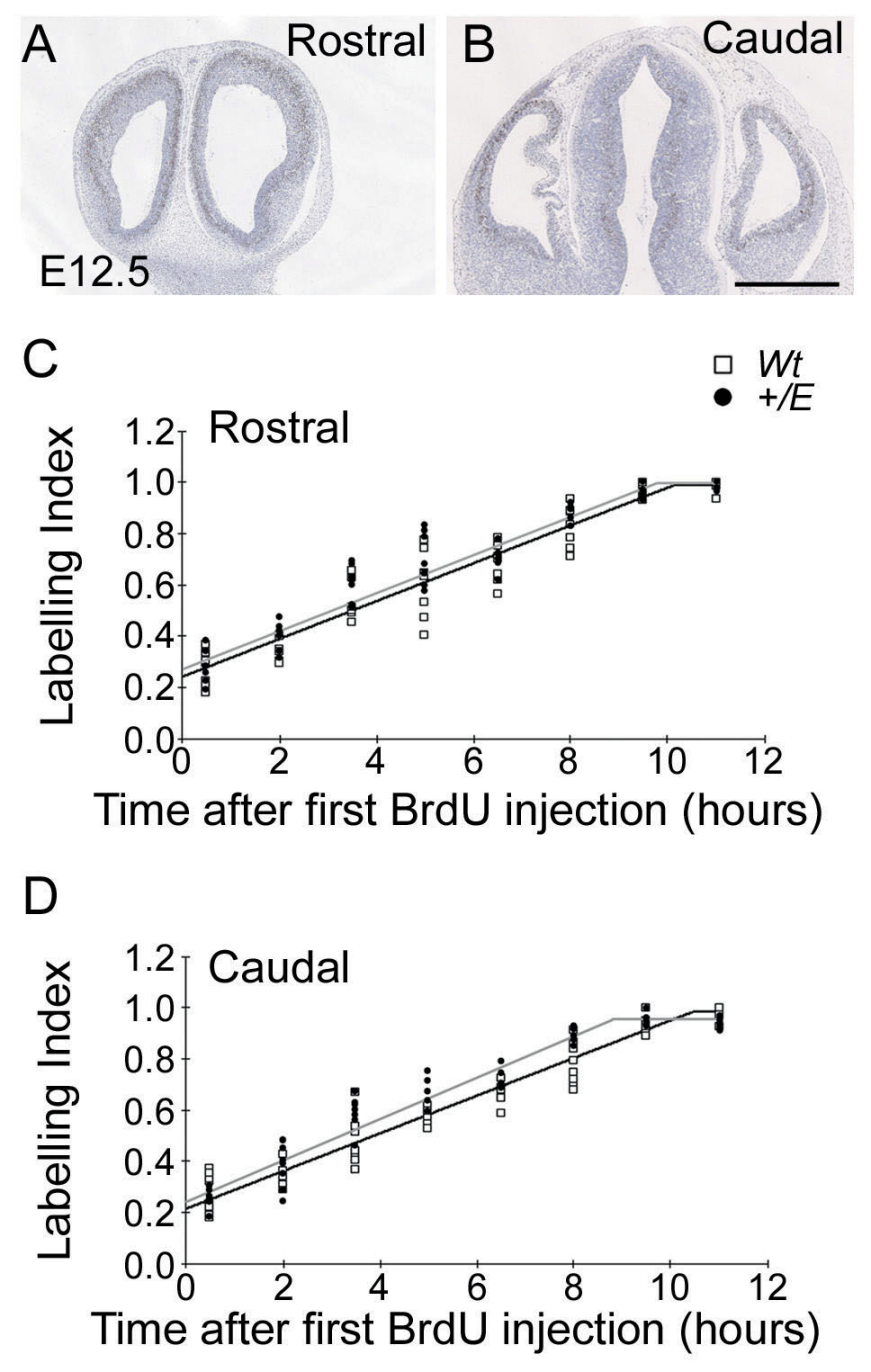
**Reduced cell cycle length in the caudal region of the *EIIa-Cre;Fgfr3*^+/*K644E *^cortical primordium at E12.5**. **(a, b) **Cumulative bromodeoxyuridine (BrdU) labeling was performed at 8 time points between 0.5 and 11 hours at E12.5. Four embryos per genotype and two counting boxes per sample were used at each time point. Panels (a, b) are representative wild-type coronal forebrain sections at the rostral (a) and caudal levels (b). Equivalent levels of sections in *EIIa-Cre;Fgfr3*^+/*K644E *^were selected using morphological landmarks. Scale bar: 670 μm. **(c, d) **LI of *EIIa-Cre;Fgfr3*^+/*K644E *^(black circles) and wild type (Wt; open squares) was plotted against each time point at the rostral (c) and caudal levels (d). In contrast to the rostral region, which showed similar profiles in *EIIa-Cre;Fgfr3*^+/*K644E *^and wild type (c), the LI plateau was reached at an earlier time point in *EIIa-Cre;Fgfr3*^+/*K644E *^compared to wild type in the caudal region (d), indicating a shorter Tc-Ts.

We have previously demonstrated the role of Fgfr3 in controlling progenitor numbers during cortical development [26,29]. In this context, we predicted that the cortical area phenotype described above might be associated with changes in cell cycle parameters along the Fgfr3 expression gradient. We therefore analyzed cell cycle kinetics in the *EIIa-Cre;Fgfr3*^+/*K644E *^cortical primordium at the onset of neurogenesis in rostral and caudal regions using the established method [42] (Figure 4a, b). The average total cell cycle length (Tc) of progenitors in the dorsal VZ is reported to be 8.1, 10.2, and 11.4 hours at E11.5, E12.5, and E13.5, respectively [43]. Based on the Fgfr3 expression gradient known to be present during E11.5-E13.5, we previously examined progenitor proliferation by the BrdU pulse labeling method at each of E11.5, E12.5, and E13.5 [26]. Increases in BrdU incorporation in the mutant VZ in comparison to the wild type were 10–20% at E11.5, while it was up to 46% at E12.5 and E13.5. In this study, we selected E12.5 for our analysis, as the difference in Tc may be too subtle to detect at E11.5.

We performed cumulative BrdU labeling over a total period of 11 hours, at 8 time points with intervals of 1.5 hours. The labeling index (LI) was then plotted at each time point after initial BrdU injection (Figure 4c, d). Four embryos per genotype and two counting boxes per sample were used at each time point. As expected, the LI progressed linearly with administration of further BrdU injections, until BrdU labeling became saturated as illustrated by the LI plateau. A similar linear progression and the time to reach the LI plateau was observed in wild type and *EIIa-Cre;Fgfr3*^+/*K644E *^at the rostral level (Figure 4c). In contrast, the LI plateau was reached at an earlier time point in *EIIa-Cre;Fgfr3*^+/*K644E *^compared to wild type in the caudal region (Figure 4d), indicating that Tc was reduced in *EIIa-Cre;Fgfr3*^+/*K644E *^compared to wild type. In contrast, the growth fraction (GF) was similar in rostral and caudal regions and in both wild type and *EIIa-Cre;Fgfr3*^+/*K644E*^, showing 99% of cortical progenitor cells proliferating at E12.5 (Table 3). This value was consistent with a previous report [43]. Interestingly, the length of S phase (Ts) was approximately 40 minutes longer in cortical progenitors in the rostral region compared to the caudal region in the wild type (Table 3). The biological significance of this variation is unknown. Comparing the wild-type and *EIIa-Cre;Fgfr3*^+/*K644E *^samples, Ts was similar at each rostral and caudal region. In contrast, Tc in the caudal region showed a striking shortening of 1.61 hours (1 hour 37 minutes) in *EIIa-Cre;Fgfr3*^+/*K644E *^compared to wild type, while that in the rostral region remained similar.

**Table 3:** Summary of cell cycle parameters

	Rostral telencephalon			Caudal telencephalon			Difference
Cell cycle parameter	Wt (h)	*+/E *(h)	%Wt	Wt (h)	*+/E *(h)	%Wt	Wt versus *+/E*
Growth fraction	0.99	0.99	100	0.98	0.96	98	
Time to reach max. LI	10.10	9.70	96	10.44	8.77	84	-1 hr 40 min
T_C_	13.32	13.31	100	13.35	11.74	88	-1 hr 37 min
T_S_	3.22	3.62	112	2.91	2.97	102	4 min
T_G2+M_				2.0	2.0	100	4 min
T_M_				0.80	0.87	109	4 min
T_G2_				1.2	1.13	94	
T_G1_				8.44	6.77	80	-1 hr 40 min

Cell cycle parameters were determined in the rostral and caudal regions of the *EIIa-Cre;Fgfr3*^+/*K644E *^(*+/E*) and wild type (Wt) littermate cortical primordium at E12.5. Four embryos per genotype and two counting boxes per sample were used at each time point. T_G1_, T_G2_, and T_M _were determined only in the caudal region where a difference in Tc was observed. Three embryos per genotype and one to two counting boxes per sample were used.

The average Tc of neuroepithelial cells is known to increase as development proceeds, mainly due to lengthening of T_G1_[43]. We further examined the individual length of G_1_, G_2 _and M phase (T_G1_, T_G2_, and T_M_, respectively) in the caudal region, where a change in Tc was observed. Three embryos were used for each genotype. With cumulative injections of BrdU, we first determined the combined length of G_2 _and M phases (T_G2+M_) based on the time taken to label all cells in the M phase (mitotic cells). T_G2+M _is known to be relatively constant and estimated to be around 2.0 hours [43]. We therefore performed single injections of BrdU for 1.5 and 2.0 hours, and then measured the percentage of phospho-histone H3^+ ^(pHH3^+^) mitotic cells that were also labeled with BrdU. At 2 hours after BrdU injection, all mitotic cells were BrdU^+ ^in both wild type and *EIIa-Cre;Fgfr3*^+/*K644E *^(n = 3). The mitotic index (MI) was similar in wild type and *EIIa-Cre;Fgfr3*^+/*K644E *^along the rostrocaudal axis (rostral, 0.061 ± 0.022 and 0.054 ± 0.016, respectively; caudal, 0.060 ± 0.019 and 0.074 ± 0.013, respectively; n = 6; not significant). Both T_M _and T_G2 _were also similar in wild type and *EIIa-Cre;Fgfr3*^+/*K644E *^(Table 3). Finally, T_G1 _was shown to be 1.67 hours (1 hour 40 minutes) shorter in *EIIa-Cre;Fgfr3*^+/*K644E *^caudal cortex progenitors compared to wild type. Therefore, the shorter Tc observed in the caudal region of the *EIIa-Cre;Fgfr3*^+/*K644E *^cortical primordium was attributed to a reduction of T_G1_.

### Cell cycle exit in the Fgfr3 mutant cortex was similar at E13.5

**Figure 5:**
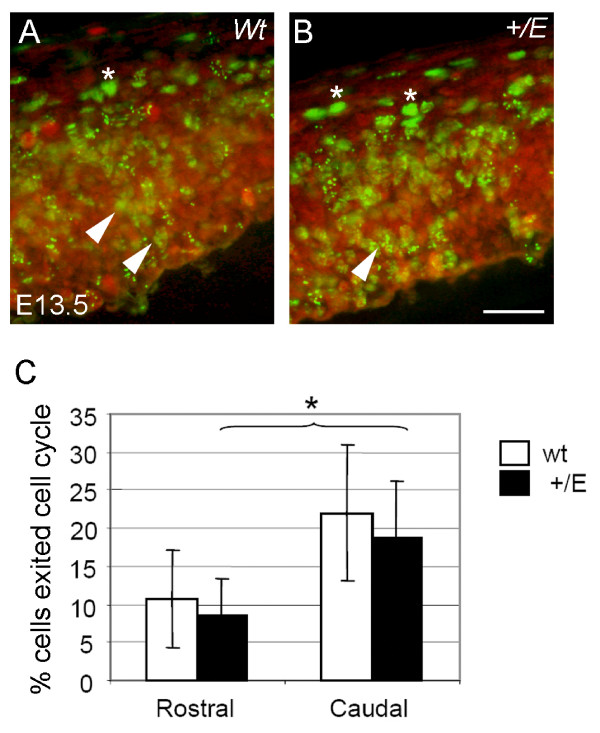
**Cell cycle exit was unchanged in the *EIIa-Cre;Fgfr3*^+/*K644E *^cortical primordium at E12.5**. **(a, b) **Double immunohistochemistry labeled bromodeoxyuridine^+ ^(BrdU^+^; green) and Ki67^+ ^(red) cells 24 hours after BrdU injection. Cells labeled with BrdU can either remain in the cell cycle (BrdU^+^Ki67^+^; yellow, arrowheads) or exit the cell cycle (BrdU^+^Ki67^-^; green, asterisks). Panels (a, b) are representative images of the dorsal region of the caudal cortical primordium in wild-type (Wt) and *EIIa-Cre;Fgfr3*^+/*K644E*^, respectively. **(c) **Cells that had exited the cell cycle were counted within a 50 μm channel in the dorsal region and their percentage was calculated by dividing the number of BrdU^+^Ki67^- ^cells by that of BrdU^+ ^cells (n = 6; *p *= 0.022, Student's *t-*test, indicated by *). Three embryos per genotype and two counting boxes per sample were used (n = 6). Scale bar: 35 μm.

In contrast, there were no significant changes in cell cycle exit in the *EIIa-Cre;Fgfr3*^+/*K644E *^cortical primordium at E13.5 (Figure 5). BrdU was injected at E12.5 and embryos were harvested after 24 hours. Double immunohistochemistry was performed to visualize BrdU^+ ^cells (those born during E12.5) and Ki67^+ ^cells (those remaining in the cell cycle). The number of cells exiting the cell cycle in the caudal region was about twofold higher compared to the rostral region in the wild type cortex (22.0 ± 9.0% versus 10.7 ± 6.3%; n = 6; not significant) and in *EIIa-Cre;Fgfr3*^+/*K644E *^(18.8 ± 7.5% versus 8.55 ± 4.9%; n = 6; *p *= 0.022). However, no significant difference was observed between wild type and *EIIa-Cre;Fgfr3*^+/*K644E *^cortices at either the rostral or caudal level (n = 6).

### Prolonged neurogenesis in the caudal Fgfr3 mutant cortex

The vast majority of cortical neurons is generated during 11 cell cycles between E11 and E18 in mice [43,44]. The shorter Tc and T_G1 _described above could result in completion of progenitor proliferation at an earlier stage, or might lead to additional cell cycles within the neurogenic phase during E11-E18. To distinguish between these possibilities, we analyzed the incorporation of BrdU in cortical progenitors at the latest stage of neurogenesis, E18.5, a time point when cortical neurogenesis is coming to completion.

**Figure 6:**
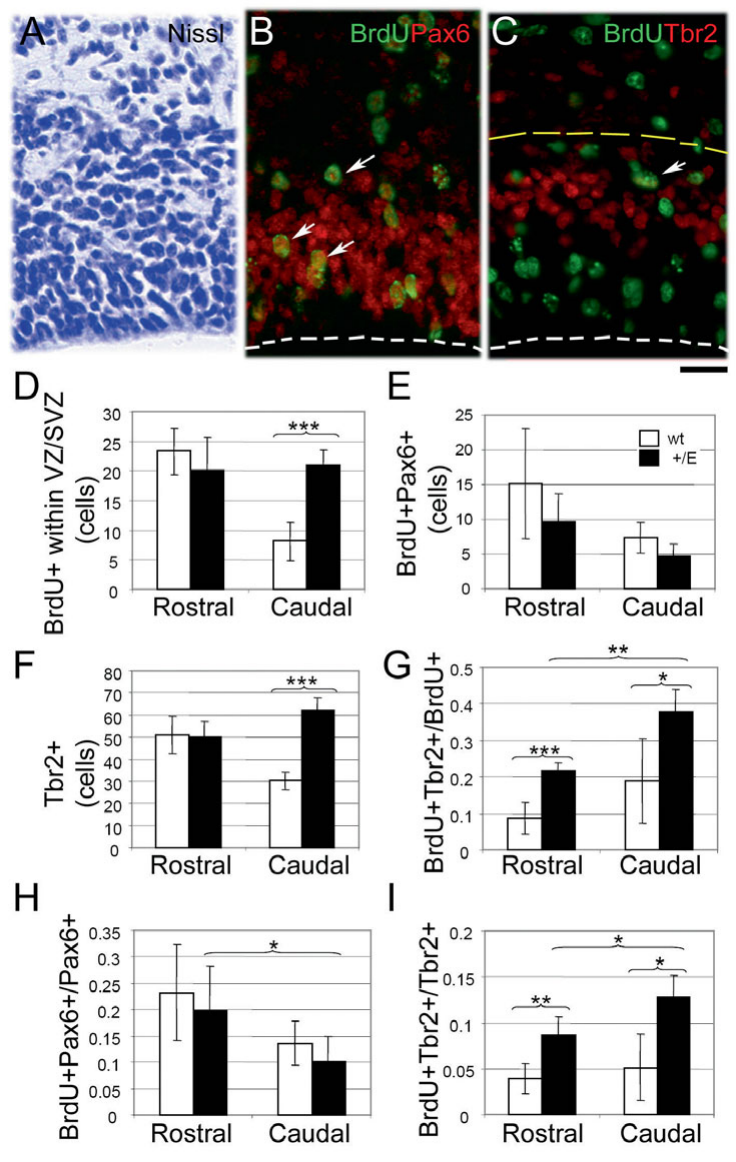
**Regulation of proliferating progenitor cells in the *EIIa-Cre;Fgfr3*^+/*K644E *^cortex at E18.5**. **(a-c) **Time-mated females were injected with bromodeoxyuridine (BrdU) for 1 hour prior to termination at E18.5. Immunohistochemistry for Pax6/BrdU (b) and Tbr2/BrdU (c) were used to identify proliferating radial glia (arrows in (b)) and intermediate progenitor cell (IPCs; arrows in (c)) on consecutive sections. Panels are examples of the staining in wild type at the rostral region. The white dashed line in (b, c) indicates the apical surface of the VZ. Cells were counted within a 100 μm channel that spans the ventricular zone (VZ)/subventricular zone (SVZ), identified by Nissl (a) and by a region containing the majority of the Tbr2 immunoreactivity (c) (marked with the yellow dashed line). Three embryos per genotype and two counting boxes per samples were used (n = 6). Scale bar: 20 μm. **(d) **Total numbers of proliferating (BrdU^+^) cells in the VZ/SVZ in the dorsal cortical regions. **(e) **Total numbers of proliferating radial glia (BrdU^+^Pax6^+^) in the VZ. **(f) **Total numbers of IPCs (Tbr2^+^) in the VZ/SVZ. **(g) **Proportion of proliferating IPCs (BrdU^+^Tbr2^+^) within the proliferating population (BrdU^+^). **(h) **Proportions of proliferating radial glia (BrdU^+^Pax6^+^) within the Pax6^+ ^population. **(i) **Proportions of proliferating IPCs (BrdU^+ ^Tbr2^+^) within the IPC (Tbr2^+^) population. Asterisks *, **, and *** indicate *p *values < 0.05, < 0.005, and < 0.005 by Student's *t-*test, respectively; See Results for details.

Although fewer than at E11-E14 [26], significant numbers of proliferating progenitor cells were still evident within the dorsal VZ/SVZ in the wild-type cortex at E18.5 (Figure 6d). Numbers of BrdU^+ ^proliferating cells were similar in rostral cortical regions in wild type and *EIIa-Cre;Fgfr3*^+/*K644E*^. In contrast, in caudal regions, the number of BrdU^+ ^cells was increased by twofold in the *EIIa-Cre;Fgfr3*^+/*K644E *^cortex compared to wild type (n = 6; *p *= 1.0 × 10^-5^). This suggests that cortical progenitors in the caudal *EIIa-Cre;Fgfr3*^+/*K644E *^cortex do not complete proliferative activity prematurely, despite a shorter Tc and T_G1_. Rather, progenitors evidently go through an increased number of cell cycles, indicating an increased proliferative capacity and prolonged neurogenic phase.

### Increase of intermediate progenitor cells in the Fgfr3 mutant cortex

The developing cortical VZ/SVZ contains two distinct populations of progenitors for glutamatergic projection neurons, known as radial glia and intermediate progenitor cells (IPCs) [45-47]. We next investigated how the two progenitor populations are differentially regulated in rostral and caudal regions of the *EIIa-Cre;Fgfr3*^+/*K644E *^cortex, using markers specific for each population, namely Pax6 (radial glia) and Tbr2 (IPCs) [47] (Figure 6 and additional file 1).

Whereas total proliferating cells in the VZ/SVZ were increased in *EIIa-Cre;Fgfr3*^+/*K644E *^in the caudal region (Figure 6d), no statistically significant differences were observed in the total number of proliferating radial glia (BrdU^+^Pax6^+ ^double-labeled) in the *EIIa-Cre;Fgfr3*^+/*K644E *^compared to the wild-type VZ (Figure 6e). In addition, the total numbers of radial glia (Pax6^+^) were not significantly different between wild type and *EIIa-Cre;Fgfr3*^+/*K644E *^at either the rostral (61.0 ± 16.1 and 46.8 ± 9.8 cells, respectively) or caudal level (48.7 ± 6.6 and 44.2 ± 5.6 cells, respectively; n = 6). In contrast, the total numbers of IPCs (Tbr2^+^) showed a twofold increase in *EIIa-Cre;Fgfr3*^+/*K644E *^compared to wild type in the caudal cortex (n = 6; *p *= 9.5 × 10^-7^), with no significant difference in rostral regions (Figure 6f). However, the proportions of proliferating IPCs (BrdU^+^Tbr2^+ ^double-labeled) as a fraction of total progenitors (BrdU^+^) were increased drastically at both the rostral (2.5-fold; n = 6; *p *= 2.6 × 10^-4^) and caudal (2-fold; n = 6; *p *= 8.4 × 10^-3^) levels in the *EIIa-Cre;Fgfr3*^+/*K644E *^cortex (Figure 6g), indicating that proliferating IPCs were increased in the *EIIa-Cre;Fgfr3*^+/*K644E *^cortex compared to wild type at both rostral and caudal regions. Finally, we examined the proliferating population of each progenitor type by quantifying proliferating radial glia and IPCs relative to the total number of each. Changes in the proportions of proliferating radial glia were not statistically significant when comparing *EIIa-Cre;Fgfr3*^+/*K644E *^and wild type cortex (Figure 6h), while proliferating IPCs increased by 2.2-fold (n = 6; *p *= 1.7 × 10^-3^) and 2.5-fold (n = 6; *p *= 1.7 × 10^-3^) in the rostral and caudal regions, respectively (Figure 6i).

In summary, the results show that the total numbers of both proliferating progenitors and IPCs were increased in the caudal cortex of *EIIa-Cre;Fgfr3*^+/*K644E *^mice, and that an increase in the proliferating IPC population was observed without a significant increase in the total progenitors in the rostral cortex of *EIIa-Cre;Fgfr3*^+/*K644E*^.

### Increased thickness in the caudal Fgfr3 mutant cortex at E18.5

The radial unit hypothesis states that the surface area of the cortex is determined early in corticogenesis by the number of radial unit founder cells generated by symmetric progenitor divisions [1]. In contrast, cortical thickness is thought to be determined by the neurogenic output from each radial unit [1], with intermediate progenitors serving to amplify, and possibly regulate, neuronal output from radial units (the radial amplification hypothesis) [48]. Previously, we showed an enlargement of surface area in the *EIIa-Cre;Fgfr3*^+/*K644E *^mutant cortical primordium compared to wild type that was visually apparent at E12.5 [29]. Based on the above theories and our previous observation, we predicted that cortical thickness should be increased in the *EIIa-Cre;Fgfr3*^+/*K644E *^mutants due to greater production or proliferation of intermediate progenitor cells.

**Figure 7:**
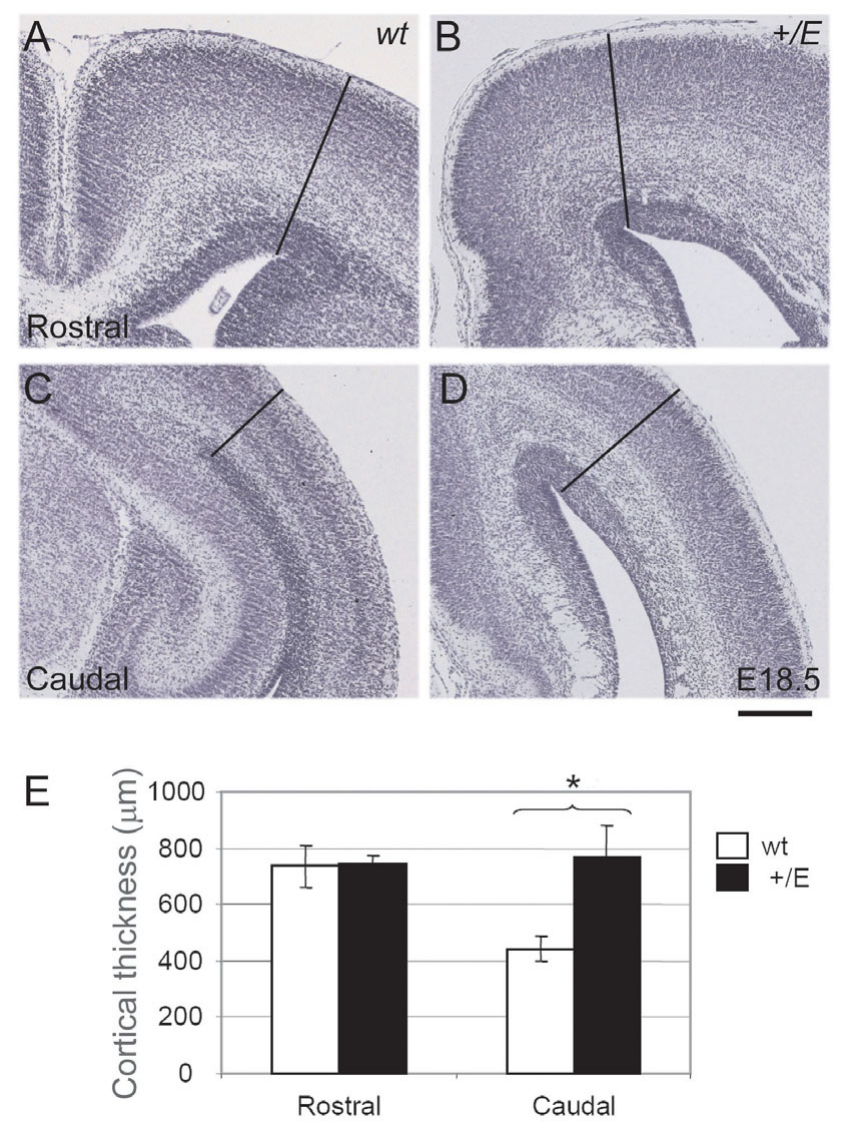
**Increased cortical thickness in the caudal *EIIa-Cre;Fgfr3*^+/*K644E *^cortex at E18.5**. **(a-d) **Representative coronal sections of the wild type (wt) (a, c) and *EIIa-Cre;Fgfr3*^+/*K644E *^dorsal cortex (b, d) at the rostral (a, b) and caudal regions (c, d) at E18.5 stained with Nissl. The black line indicates an example of the measurements taken. **(e) **Quantification of cortical thickness, comparing the *EIIa-Cre;Fgfr3*^+/*K644E *^cortex and wild type (n = 3; *p *= 0.042, Student's *t-*test, indicated by *). Scale bar: 330 μm.

We measured cortical thickness at early and late stages of neurogenesis. At E12.5, the cerebral wall consists mostly of the VZ with a small area of SVZ starting to appear, particularly in the lateral region, which is apparent with Tbr2 immunoreactivity [45,47]. There were no significant differences in cortical thickness between the wild type and *EIIa-Cre;Fgfr3*^+/*K644E *^mutant at either the rostral or caudal level (data not shown), which is consistent with our previous report where thickness was measured at a medial level [29]. At E14.5, the presence of the SVZ is more apparent, with a concordant increase in Tbr2^+ ^cells, and the cortical plate starts to emerge [45,47]. We have indeed shown previously that at E14.5, the cortical thickness was increased in the *EIIa-Cre;Fgfr3*^+/*K644E *^mutant by 38.1% (medial level) [29]. In contrast, at E18.5, more distinct laminar structures emerge in the cortical plate, although they are not yet mature. An increase in cortical thickness in *EIIa-Cre;Fgfr3*^+/*K644E *^compared to wild type was visually apparent at the caudal level, particularly that of the intermediate zone and the cortical plate (Figure 7c, d). Our analysis shows that the thickness of the caudal cortex was increased by 74% in the *EIIa-Cre;Fgfr3*^+/*K644E *^mutant compared to wild type embryos (n = 3; *p *= 0.042), but rostral cortex thickness was not significantly changed (Figure 7e). This regional difference likely reflects the significant increase of IPC numbers, and prolonged neurogenesis observed in the *EIIa-Cre;Fgfr3*^+/*K644E *^caudal cortex (Figure 6).

## Discussion

**Figure 8:**
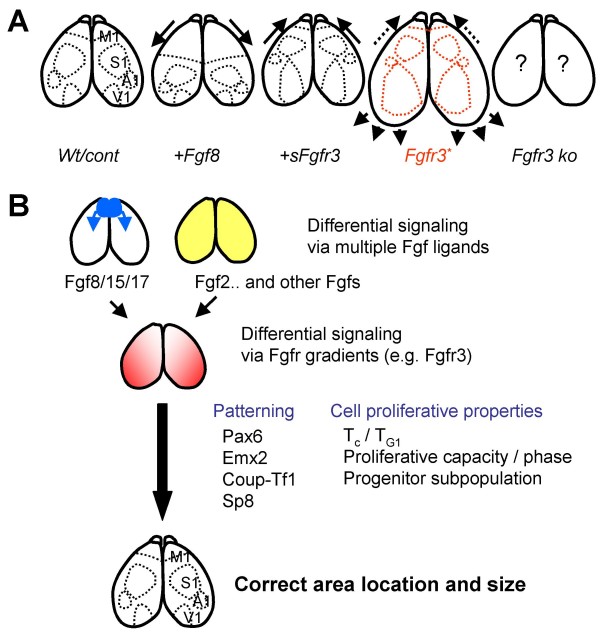
**A model for the mechanisms of cortical area formation regulated by Fgf signaling**. **(a) S**ummary of cortical area positioning upon manipulation of Fgf signaling. Anterior over-expression of Fgf8 caudally shifts cortical areas, while sequestration of Fgf8 activity by ectopic expression of a truncated Fgfr3 protein (sFgfr3) leads to a rostral shift [6]. In the current study, a genetic model of biochemically active Fgfr3 kinase domain mutant (Fgfr3*) showed a 'rostral' shift of cortical areas, likely to have resulted from a massive surface area expansion selectively occurring in caudal cortical areas. The area positions of Fgfr3 knockout (ko) mice are currently unknown. Cont, control; Wt, wild type. **(b) **We hypothesize that, being expressed in a unique graded pattern at the outset of neurogenesis, Fgfr3, together with other Fgf receptor members, may regulate formation of cortical areas, mediating signals from multiple Fgf ligands with distinct functions and diverse expression patterns. This study indicates that various proliferative parameters, including Tc (namely T_G1_), proliferative capacities of the cortical progenitors, and radial glia and intermediate progenitor cell populations, could be regulated along the Fgfr3 expression gradient. Therefore, Fgf signaling regulates cortical area formation by controlling both patterning and cell proliferative properties.

In the present study, we demonstrate that genetic models with biochemically activating kinase-domain mutations in *Fgfr3 *(*Nestin-Cre;Fgfr3*^+/*K644E *^and *Foxg1-Cre;Fgfr3*^+/*K644M*^) show a massive surface area expansion of the caudolateral cortex, which is highly correlated with the gradient of Fgfr3 expression in the VZ at early stages of neurogenesis (summary in Figure 8a). Is the cortical phenotype of the current Fgfr3 mutant model caused by a change in patterning or by size expansion of the caudal region? Little effect was observed on frontal area size (Figures 1 and 2, and Tables 1 and 2) or transcription factor gradients in the mutant cortex (Figure 3), suggesting that cortical patterning was not significantly influenced by increased Fgfr3 activity. In contrast, shortening of Tc and T_G1 _(Figure 4 and Table 3) at E12.5, and augmentation of BrdU incorporation at E18.5 (Figure 6) were observed selectively in the caudal region of the Fgfr3 mutant cortex, supporting the idea that the relative 'rostral shift' resulted from size expansion of the caudal region mostly owing to the cell proliferative effects.

### Regulation of patterning by Fgfr3

The expression of the rostral signaling factor Fgf8 and protomap transcription factors, including Pax6, Emx2, and Coup-Tf1, showed no obvious changes in mice with kinase-domain mutations in Fgfr3 (*EIIa-Cre;Fgfr3*^+/*K644E*^) at E9.5 and E11.5-E13.5, respectively (Figure 3). Interestingly, it was also reported that, despite the loss of the most anterior structure of the forebrain, the changes in gradients of Emx2 and Pax6 expression were relatively subtle in Fgfr1 knockout mice (*Foxg1-Cre;Fgfr1*^*flox*/*flox*^) [17]. These observations are in contrast to results of Fgf8 over-expression by *in utero *electroporation [24,41] and in genetic models with reduced Fgf8 [7,8], where graded expression of transcription factor genes was visibly shifted according to the changes in Fgf8 signaling. Genes that define the protomap have been intensively screened [4]. It is possible that genes other than Pax6, Emx2, and Coup-Tf1, are regulated in the current model. For example, transcription factor Sp8 was recently shown to play a role in cortical area patterning [49,50].

### Regulation of neurogenesis by Fgfr3

In our previous study, we used 1 hour BrdU incorporation as a measure of cell proliferation in the cortical VZ/SVZ in the *EIIa-Cre;Fgfr3*^+/*K644E *^developing cortex at E11.5, E12.5 and E13.5 [26]. Graded BrdU incorporation was observed with the highest increase in the mutant cortex compared to wild type in the caudal region at E12.5 and E13.5. Although overall cell proliferation became less profound as development progressed [43], the magnitude of the proliferation increase in the mutant progenitor population compared to that of wild type became larger. In the current study, we addressed how cell cycle parameters, including cell cycle length and cell cycle exit, are regulated by Fgfr3 early in neurogenesis. We have shown that Fgfr3 activation in caudal regions of the developing forebrain reduces cell cycle duration (Figure 4 and Table 3). Based on the cell cycle length hypothesis, whereby shorter cell cycle length is associated with proliferative rather than neurogenic division [51], we expected that the shorter cell cycle length observed in this study would result in an overall reduction of cells exiting the cell cycle. However, cell cycle exit was not affected by activation of Fgfr3. A shift in the subpopulations of progenitors within the VZ could also influence the overall measurement of cell cycle duration. However, at E12.5, when we performed the analysis, the cortical wall consisted mainly of radial glia, with only a few IPCs present [45,47]. Therefore, little influence from the IPC population is expected. Indeed, little difference in the balance of these progenitor populations was observed at E12.5 (data not shown).

In contrast, an increase in the progenitor subpopulation was observed at E18.5 at the very late stage of neurogenesis (Figure 6). The total number of proliferating progenitors, and the number of IPCs were increased in the caudal cortex of *EIIa-Cre;Fgfr3*^+/*K644E *^mice, indicating the prolongation of neurogenesis upon activation of Fgfr3. In this study, we focused on the effect of Fgfr3 activation in neurogenesis. Tbr2^+ ^progenitors are known to be glutamatergic in all systems studied to date, including the developing cortex [52]. In Tbr2-Gfp mice, which provide short-term lineage tracing, GFP has never been detected in glia, but only in neurons (T Kowalczyk and R Hevner, manuscript submitted). However, Fgfr3 expression has been reported in glial populations at postnatal stages [53,54]. Therefore, it cannot be excluded that a small proportion of glia contributed to the increase in BrdU incorporation observed at E18.5 (Figure 6).

Increases in cortical thickness was observed specifically in the caudal region in *EIIa-Cre;Fgfr3*^+/*K644E *^embryos (Figure 7). Increases in the thickness of the intermediate zone likely represent radially migrating late-born neurons resulting from prolonged neurogenesis (Figure 6). The timing of cell cycle exit is closely linked to laminar fate, and growing evidence suggests that the upper-layer neurons are contributed by the SVZ, or IPC, progenitor population [55,56]. Given the increase in Tbr2^+ ^progenitors in our mutant mice observed at E18.5, it would be interesting to examine whether upper neurons are preferentially increased in the postnatal cortex in Fgfr3 mutant models. Despite the apparent similarities in Fgfr3 expression levels in radial glia and IPC populations, how Fgfr3 activation preferentially influences Tbr2^+ ^progenitor proliferation at E18.5 is also an intriguing question, which needs to be addressed in the future.

The study indicates that Coup-Tf1 functions to increase overall neurogenesis by promoting cell cycle exit at early to mid-stages of neurognesis (E11.5 and E15.5) and that suppression of MAPK and activation of β-catenin signaling pathways are responsible for this effect [57]. Interestingly, the study also shows that Coup-Tf1 promotes Fgfr3 expression, making Fgfr3 an interesting downstream target and possibly an effecter of cortical patterning, translating the gradients of transcription factors into progenitor proliferation. However, at this moment this seems to be unlikely as we have shown in this and previous studies [29] that activation of Fgfr3 leads to an increase in cortical progenitor proliferation, little effect on cell cycle exit, and activation of the MAPK signaling pathway.

### Fgfr3 activation as a cause of human cortical malformation

The current study implicates the importance of FGFR3 in the normal and pathological development of the cerebral cortex in humans. Occipitotemporal surface expansion and hippocampal dysplasia are two prominent characteristics of the cortical malformation in human TD, a disease caused by kinase-domain mutations in human FGFR3 corresponding to the models used in this study [34]. Mice do not normally form sulcus, which is a known limitation of the animal model. However, the selective surface area expansion in the caudal cortical area observed in the mouse model (Figures 1 and 2) is likely to reflect premature gyrification of the occipitotemporal cortex in human TD. And the study supports the hypothesis that human TD pathology results from an early regional increase of surface area [34]. In addition, the reduced expression of a cortical hem marker, Wnt2b, was observed in the *EIIa-Cre;Fgfr3*^+/*K644E *^cortical primordium (Figure 3). The increase in Fgf signaling via activation of Fgfr3 may suppress growth of the hem and/or its differentiation. Indeed, our preliminary observations of the current mouse model revealed hippocampal dysplasia with a visibly apparent reduction in size of CA3/dentate gyrus (data not shown). The hippocampus phenotype of Fgfr3 mutant models and its mechanism is to be addressed in a separate report.

Further clarification of Fgfr3 functions in cerebral cortex development will depend on detailed analysis of the Fgfr3 knockout cortex. Results from characterization of cortical patterning in Fgfr3 knockout mice have not been conclusively reported so far [28]. In our hands, the Fgfr3 knockout mice [27] rarely survive beyond P1 in the C57/Bl6 background (data not shown). Further investigation of the Fgfr3 knockout phenotype, possibly in a more favorable genetic background, must be undertaken.

### Regulation of cortical area location and size by Fgf signaling

We hypothesize that both patterning and progenitor proliferation are regulated by signaling from the multiple Fgf ligands, possibly utilizing the graded Fgfr3 activity and that of the other Fgf receptor members present in the cortex (Figure 8b). Different Fgfs could define positional information within the cortex by exerting their effects in combination. The Cooperative Concentration Model [4] suggests that area identities are specified by the presence of multiple transcription factors at different concentrations, rather than by sharply bordered expression of certain genes. Despite overlapping expression patterns of Fgfr1, Fgfr2, and Fgfr3 in the cortical primordium, the effect of the *Fgfr3 *knockout turned out to be surprisingly localized and specific. Deletion of Fgfr1 (*Foxg1-Cre;Fgfr1*^*flox*/*flox*^) did not result in total loss of rostro-caudal patterning in the cortex, but resulted in a milder change in patterning of gene expression, with a loss of the most anterior telencephalic structures, including the olfactory bulbs [17]. Similarly, despite the graded expression pattern of Fgfr3 across the rostrocaudal axis, a selective expansion of the caudal cortex was observed upon activation of Fgfr3 in this study, supporting the Cooperative Concentration Model.

*In vitro *assays showed that the Fgfr3IIIc isoform, the main alternative splicing isoform in the developing brain [58], is able to mediate signals from Fgf2, Fgf8, Fgf15, Fgf17, and Fgf18 with high affinity [15,22]; however, binding of Fgf7 produced very little signal [15]. It is theoretically possible that Fgfr3 could mediate the effects of Fgf2/8/15/17/18, depending on the ligand expression domains and its own expression, exerting its effect either in patterning and/or in progenitor proliferation. The results of this study, however, indicate that Fgfr3 is unlikely to mediate the rostral Fgf signals in patterning, but rather mediates the effect in progenitor proliferation in the caudal region. In contrast, the rostral Fgf signals are likely to be mediated by Fgfr1 and Fgfr2 [17,19]. The ligand that utilizes Fgfr3 in the caudal region remains unknown.

## Conclusion

We demonstrate that activation of Fgfr3 selectively promotes growth of the caudolateral (occipitotemporal) cortex, similar to the cortical malformation observed in human TD. We provide evidence that activation of Fgfr3 alters caudal-specific cell proliferative properties, namely shortening of the cell cycle length and prolongation of neurogenesis, without altering cell cycle exit. Few changes were observed in early patterning events. Being expressed in a unique graded pattern at the outset of neurogenesis, Fgfr3, together with other Fgf receptor members, may regulate formation of cortical areas.

## Materials and methods

### Mice

All procedures were performed in accordance with the Project Licence under Home Office Animal (Scientific Procedures) Act 1986. Mice heterozygous with the Fgfr3 mutation and with one copy of the *Cre *gene, including *EIIa-Cre;Fgfr3*^+/*K644E*^, *Nestin-Cre;Fgfr3*^+/*K644E *^and *Foxg1-Cre;Fgfr3*^+/*K644M*^, were produced by crossing either *Fgfr3*^+/*K644Eneo *^[30] or *Fgfr3*^+/*K644Mneo *^[31] with *EIIa-Cre *[30], *Nestin-Cre *[36], or *Foxg1-Cre *[37], respectively. *Fgfr3*^+/*K644Eneo*^, *Fgfr3*^+/*K644Mneo*^, and *Nestin-Cre *colonies were maintained in C57BL/6. The backgrounds of *EIIa-Cre *and *Foxg1-Cre *were 129;FBV/N and 129Svj, respectively. Animals were genotyped using the K644 mutation site [30] and the presence of Cre. In all experiments, littermates with genotypes of *+/+; Fgfr3*^+/+^, *+/cre; Fgfr3*^+/+^, or *+/+; Fgfr3*^+/*K644E*/*Mneo*^, were used as controls.

### Analysis of the cortical area position

Mice were perfused by 4% (w/v) paraformaldehyde in phosphate-buffered saline (PBS). After cryoprotection, cortices were flat-frozen using the weight of a microscopic slide. Tangential sections (50 μm) were subjected to immunohistochemistry with anti-mouse 5-hydroxy-tryptamine (5-HTT; 1:2000; Calbiochem, Nottingham, UK) [59] or cytochrome C oxidase histochemistry [38]. Sections were incubated in reaction mix containing 40 mg/ml sucrose, 0.15 mg/ml cytochrome C (C-2506; Sigma, Dorset, UK), 0.5 mg/ml DAB (D-3001, Sigma) in 0.1 M phosphate buffer, at 37°C for 3 hours to overnight. The measurement of the barrel location was performed by Image J (NIH).

### Cumulative BrdU labeling

Average length of total cell cycle duration (Tc), length of the S phase (Ts), and growth fraction (GF) were determined according to published protocols [43,60,61]. Mice were time-mated and the day of the vaginal plug was counted as E0.5. At E12.5, mice were injected intraperitoneally with BrdU in PBS at 50 μg/g body weight. Subsequent injections were performed at a maximum interval of 3 hours, with a final BrdU injection 0.5 hour prior to termination. Embryos were collected at 8 survival points, 0.5, 2.0, 3.5, 5.0, 6.5, 8.0, 9.5 and 11.0 hours after the initial injection. Morphological landmarks based on plates 1 and 6 at E12.5 [62] were used to identify sections at the rostral and caudal levels, respectively. A 25 μm channel spanning the dorsal VZ was used for counting BrdU-labeled cells. The LI was calculated as the proportion of BrdU-labeled cells to total cells. A least-squares curve fit was generated in the graphs of LI versus survival time post-BrdU injection using a Microsoft Excel spreadsheet kindly provided by Professor Richard Nowakowski [42]. The GF was calculated as the maximum LI value attained over the experimental period. Tc and Ts were calculated using the following equations [61]: *y*-intercept = GF × Ts/Tc; Time to reach maximum LI = Tc - Ts.

To determine the combined lengths of G_2 _and M (T_G2+M_) phases, a single-injection BrdU-labeling protocol was used [61]. T_G2+M _was calculated as the length taken to label all cells in the M phase (mitotic cells) with BrdU. Within each 100 μm channel in the dorsomedial cortex, the percentage of BrdU^+^pHH3^+ ^cells was determined. MI was calculated by dividing the number of mitotic cells by the total number of cells within a 50 μm channel in the dorsomedial cortex. The duration of M phase (T_M_) was then calculated using the following equations: MI = Total number of mitotic cells/Total number of cells; T_M _= Tc × MI. T_G2 _and T_G1 _were calculated using the following equations: T_G2 _= (G_2 _+ M) - T_M_; T_G1 _= Tc - Ts - (T_G2+M_).

### Immunohistochemistry

Coronal sections (10 μm) were deparaffinised and rehydrated. Antigen retrieval was achieved by boiling in 10 mM citrate buffer (pH 6.4) for 30 minutes. Endogenous peroxidase activity was quenched by 3% (v/v) hydrogen peroxide for 10 minutes. Blocking was in 10% normal goat serum in PBS. Incubation with the anti-BrdU antibody (B-44, 1:75; BD Biosciences, Oxford, UK) was performed overnight at 4°C. The secondary antibody was applied for 1 hour (mouse Immpress Kit, VectorLabs, Peterborough, UK). Immunoreactivity was detected by 3,3'-diaminobenzidine (DAB) and counterstained in haematoxylin Harris (Surgipath, Peterborough, UK). For mitotic index, pHH3 rabbit polyclonal antibody (1:200; Upstate, Millipore, Herts, UK) and rabbit secondary antibody (rabbit Immpress Kit, VectorLabs) was used. For double immunohistochemistry, sections were incubated in 2 mol/dm^3 ^HCl for 1 hour at 37°C after antigen retrieval. Blocking solution was 10% (v/v) normal goat serum, 2% bovine albumin, 0.1% (v/v) Triton-X100 in PBS. Antibodies used were: anti-Tbr2 (rabbit polyclonal, 1:2000; Chemicon, Nottingham, UK); anti-Pax6 (rabbit polyclonal, 1:500; Covance, Cambridge BioScience, Cambridge, UK); anti-Ki67 (rabbit polyclonal, 1:1000; Novocastra, New Castle upon Tyne, UK); goat anti-mouse AlexaFluor 488, and goat-anti-rabbit AlexaFluor 594 (1:200; Invitrogen, Paisley, UK). Sections were mounted in VectaSield (VectorLabs) containing 4',6-diamidino-2-phenylindole (DAPI).

### *In situ *hybridization

Riboprobe templates were kindly provided by Drs Gail Martin (Fgf8), John Rubenstein (Coup-Tf1), David Price (Pax6), Shinichi Aizawa (Emx2), and Wnt2b (Thomas Theil). Embryos were fixed in 4% (w/v) paraformaldehyde/PBS overnight. Embryos were dehydrated and stored in methanol at -20°C until genotyped. Embryos were rehydrated in methanol/PBS with 0.1% (v/v) Tween-20 (PTW). Treatment with 10 μg/ml Proteinase K/PTW was performed at room temperature for exactly 5 (E9.5) or 15 minutes (E11.5 and E13.5) and the reaction was stopped by incubation in 4% (w/v) paraformaldehyde, 0.1% glutaraldehyde in PBS. Embryos pre-incubated in hybridization buffer (50% (v/v) deionized formamide, 5 × SSC, 0.8% (v/v) Tween-20, 50 μg/ml heparin, 1 mg/ml yeast tRNA, 5 mM EDTA, 0.1% (w/v) CHAPS, 2% (w/v) Blocking Reagent, Roche, Welwyn, UK) at 65°C for 1 hour. Riboprobes were diluted 1:300 in the hybridization buffer and denatured at 80°C for 10 minutes. Hybridization was performed at 65°C overnight. Embryos were washed with post-hybridization buffer (50% (v/v) formamide, 1 × SSC, 0.1% (v/v) Tween-20) at 65°C, then with Tris-buffered saline (TBS) with 0.1% (v/v) Tween-20 (TBST) at room temperature. Embryos were blocked in 2% (w/v) Blocking Reagent solution (Roche) with 20% (v/v) Sheep serum (S2263, Sigma) for 2 hours before incubation with anti-DIG antibody (1:1500; Roche) at 4°C overnight. After washes with TBST, color reaction was performed with Nitroblue tetrazolium (NBT) and 5-bromo-4-chloro-3-indolyl phosphate (BCIP) in NTMT (100 mM Tris, pH 9, 100 mM NaCl, 50 mM MgCl_2_, 1% (v/v) Tween-20).

### Cortical thickness

Comparable regions of *EIIa-Cre;Fgfr3*^+/*K644E *^and wild type littermates were selected using morphological landmarks consistent with plates 4 and 12 at E18.5 [62]. Using Image J, five lines were drawn from the ventricular to the basal surface and an average was calculated from the length measured. Three embryos were used per genotype.

### Statistics

Student's *t*-test was performed to test the significance of difference in numerical data for two-sample unequal variance with a two-tailed distribution. A standard deviation is used for presentation of data.

## Abbreviations

BrdU: bromodeoxyuridine; E: embryonic day; Fgf: fibroblast growth factor; Fgfr: Fgf receptor; GF: growth fraction; IPC: intermediate progenitor cell; LI: labeling index; MI: mitotic index; P: postnatal day; PBS: phosphate-buffered saline; pHH3: phospho-histone H3; SVZ: subventricular zone; TD: thanatophoric dysplasia; VZ: ventricular zone.

## Competing interests

The authors declare that they have no competing interests.

## Authors' contributions

RET designed, performed and analyzed experiments for cell cycle parameters, proliferative phase and cortical thickness. MLE and ACF helped with these experiments. PCK, NAG, JK, CSM performed cortical area analysis. RFH helped with the interpretation of results and writing of the final draft. TI coordinated the project, designed and performed the experiments, analyzed and interpreted the results and wrote the paper. All authors approved the manuscript.

## Supplementary Material

Additional file 1 **Increase of proliferating intermediate progenitor cells in the *EIIa-Cre;Fgfr3*^+/*K644E *^cortex at E18.5**. (a-d) Time-mated females were injected with bromodeoxyuridine (BrdU) for 1 hour prior to termination at E18.5. Immunohistochemistry for Tbr2 (red) and BrdU (green) were used to identify proliferating intermediate progenitor cells (IPCs) in wild-type (a, c) and *EIIa-Cre;Fgfr3*^+/*K644E *^cortex (b, d) in the rostral (a, b) and caudal regions (c, d). Scale bar: 32 μm. Click here for file
