# Supplementary figures and images for: Fgf receptor 3 activation promotes selective growth and expansion of occipitotemporal cortex
Source: Neural Dev. 2009 Feb 3;4:4. doi: 10.1186/1749-8104-4-4 (PMC2661882; doi:10.1186/1749-8104-4-4)

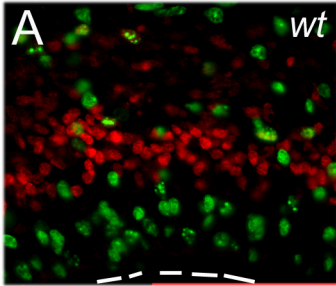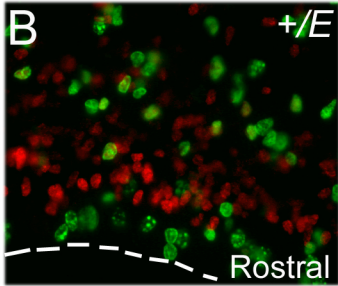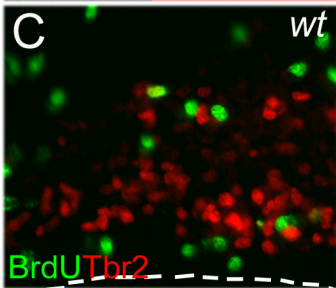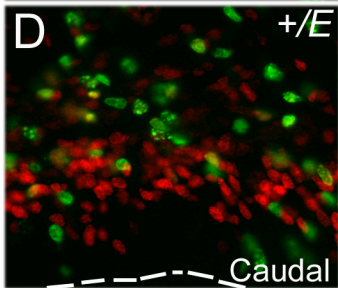

Supplement: Additional file 1 — Increase of proliferating intermediate progenitor cells in the EIIa-Cre;Fgfr3+/K644E cortex at E18.5. (a-d) Time-mated females were injected with bromodeoxyuridine (BrdU) for 1 hour prior to termination at E18.5. Immunohistochemistry for Tbr2 (red) and BrdU (green) were used to identify proliferating intermediate progenitor cells (IPCs) in wild-type (a, c) and EIIa-Cre;Fgfr3+/K644E cortex (b, d) in the rostral (a, b) and caudal regions (c, d). Scale bar: 32 μm. [file 1749-8104-4-4-S1.pdf]
